# Supplementary material for: Strong and specific associations between cardiovascular risk factors and white matter micro- and macrostructure in healthy aging
Source: Neurobiol Aging. 2019 Feb;74:46–55. doi: 10.1016/j.neurobiolaging.2018.10.005 (PMC6338676; doi:10.1016/j.neurobiolaging.2018.10.005)
Supplement: Supplementary Material [file mmc1.pdf]

Supplementary Material

Supplementary Methods: MRI data acquisition and pre-processing

Data were acquired at the MRC Cognition and Brain Sciences Unit using a Siemens 3T TIM TRIO (Siemens, Erlangen, Germany) and a 32-channel head coil. A T1-MPRAGE sequence was used to acquire anatomical data at 1mm^3 isotropic resolution (TR: 2250ms; TE: 2.98ms; TI: 900ms; 190Hz; FOV: 256 × 240 × 192mm; flip angle: 9°; GRAPPA acceleration factor: 2; acquisition time: 4m 32s). Diffusion weighted images (DWI) were acquired with a twice-refocused spin echo sequence, optimized for the estimation of the diffusion kurtosis tensor and associated metrics. Thirty diffusion gradients were used for each of the two b-values: 1000 s/mm^2 and 2000 s/mm^2, and three images were acquired with a b-value of 0 (Other parameters — TR: 9100 ms; TE: 104 ms; voxel size: 2 mm^3 isotropic; FOV: 192 × 192mm; axial slices: 66; number of averages: 1; acquisition time: 10m 2s).
All MRI data were processed using a combination of SPM12 software (www.fil.ion.ucl.ac.uk/spm) and in-house code implemented in the AA batching software. See Taylor et al. (2017) for a complete description of the pipeline.

Anatomical T1 and T2 images were co-registered to an MNI space template and segmented into 6 tissue classes: GM (grey matter), WM (white matter), CSF (cerebrospinal fluid), bone, soft tissue, and residual noise. Diffeomorphic registration (DARTEL) spatial normalization was performed in SPM12. DARTEL was applied to GM images to create a group template, which was then transformed once more to MNI space. Changes in volume due to spatial deformation were accounted for by modulating the images using the Jacobean of the deformation field, resulting in voxel-wise estimates of brain GM volume. DARTEL is a template-based approach particularly suited to assessing global morphology. DARTEL incorporates both GM and WM segmentations in estimating the high-dimensional flow-fields, which has been shown to increase registration accuracy compared to other non-linear techniques (de Groot et al., 2013).

Diffusion weighted images were first registered to the individual's T1 image and skull stripped using the FSL brain extraction tool (http://fsl.fmrib.ox.ac.uk/fsl/fslwiki/). FA, MD and MK were then extracted from the data. MK was estimated by linear fitting of a higher-order tensor using in-house code (Neto Henriques et al., 2015). The resulting images from these two pipelines were spatially normalized using the spatial deformation field obtained from the DARTEL normalization step above, and modulated by the Jacobean of the deformation field.

Fluid attenuation inversion recovery (FLAIR) images were acquired on the same scanner (TR: 9000ms; TE: 100ms; TI: 2500ms; FOV: 220mm; Flip Angle: 150deg; Slice Thickness: 4mm; Orientation: Transverse). Raw FLAIR DICOM images were processed in a separate pipeline using the LST toolbox for SPM (Schmidt et al., 2012).

Supplementary Analyses: Testing for potential confounds

We carried out a series of supplementary analyses of white matter lesion burden and MD to examine whether our results could be explained by possible confounders such as sex differences, medication, cardiovascular risk-factors or social and lifestyle factors.

Sex differences. To examine whether possible sex differences confounded our results, we ran multi-group models and imposed increasingly restrictive invariance constraints across sexes. These equality constraints test the hypothesis that although the mean scores may differ across groups (e.g. poorer overall cardiovascular health in men), the association between cardiovascular health and white matter outcomes is the same across sexes. For both lesion burden and MD, we found no statistical differences between a model with configural and weak (lesion burden: $\Delta\chi^2(9) = 8.70$, $p = .465$; MD: $\Delta\chi^2(9) = 8.56$, $p = .479$), and weak and strong invariance constraints across sexes (lesion burden: $\Delta\chi^2(9) = 0.32$, $p > .999$; MD: $\Delta\chi^2(17) = 0.05$, $p > .999$), suggesting that a model with strong invariance constraints was most parsimonious. This indicates that the overall model structure, factor loadings and intercepts did not differ significantly between males and females.

Antihypertensive medication. The same approach of testing invariance constraints across groups was taken to test for differences between participants taking or not taking antihypertensive medication. Again, we found evidence for strong invariance for both lesion burden and MD. We found no statistical differences between a model with configural and weak (lesion burden: $\Delta\chi^2(9) = 11.21$, $p = .262$; MD: $\Delta\chi^2(9) = 12.68$, $p = .178$), and weak and strong invariance constraints across medicated and unmedicated participants (lesion burden: $\Delta\chi^2(9) = 0.25$, $p > .999$; MD: $\Delta\chi^2(17) = 0.03$, $p > .999$), suggesting that the pattern of results did not differ significantly between these groups.

Cardiovascular risk factors. To test whether cardiovascular risk factors like diagnosed hypercholesterolemia etc. (see Table 1 for full list) influenced our results, we tested invariance across participants with and without diagnosed cardiovascular risk factors. We found no statistical

differences between a model with configural and weak (lesion burden: $\Delta\chi^2(8) = 6.89$, $p = .549$; MD: $\Delta\chi^2(9) = 7.60$, $p = .575$), and weak and strong invariance constraints across these groups (lesion burden: $\Delta\chi^2(8) = 0.13$, $p > .999$; MD: $\Delta\chi^2(17) = 0.08$, $p > .999$), suggesting that the pattern of results was similar in participants with and without known cardiovascular conditions.

Social and lifestyle factors. To test whether social class, education levels, smoking and alcohol consumption confounded results, we re-ran models including covariates for these four variables. The results changed very little - all associations retained their directionality and significance level (Supplementary Tables 17 and 18).

Supplementary Figures

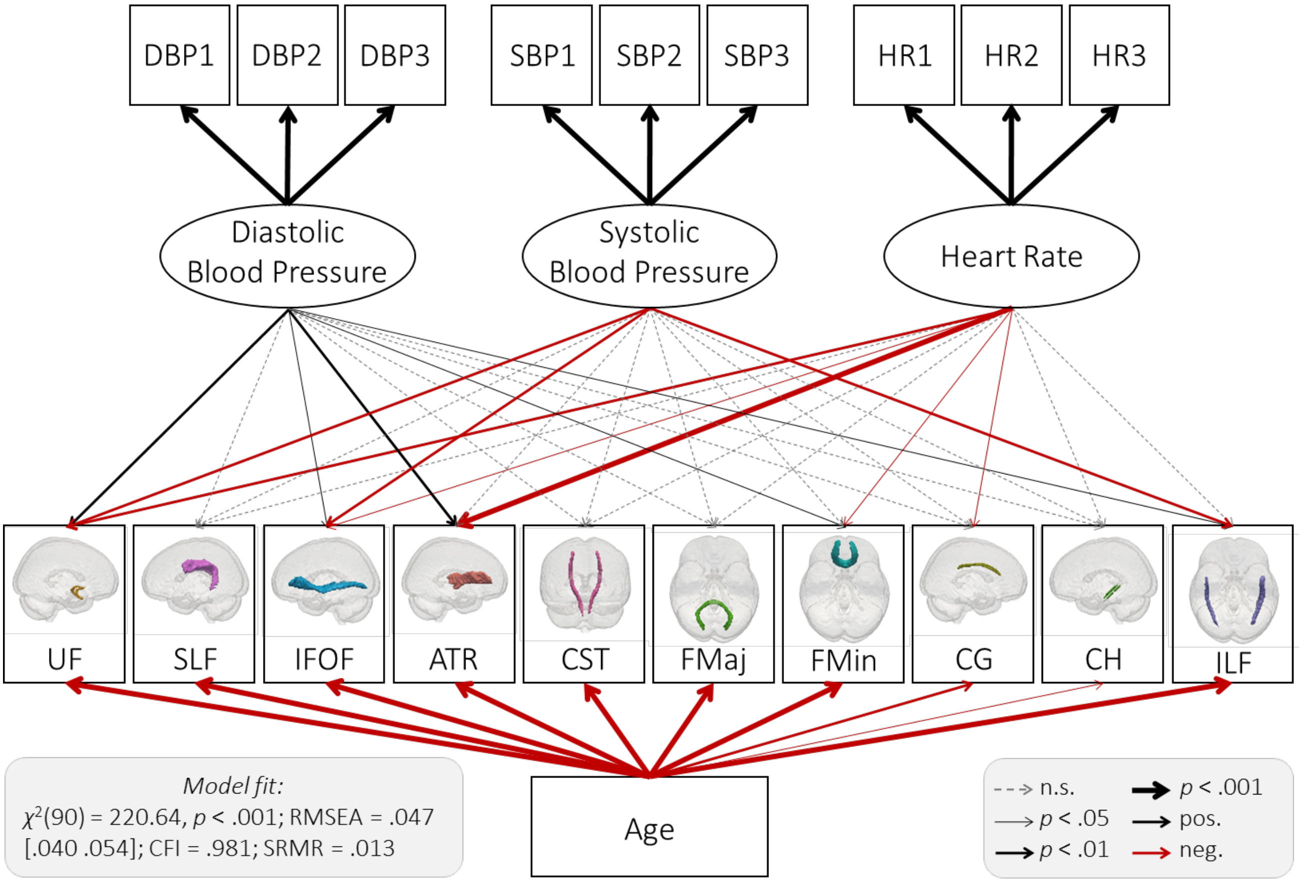

Supplementary Figure 1. Path Model of the Relationship between Cardiovascular Health and FA. Diastolic blood pressure, systolic blood pressure and heart rate were modelled as latent variables (Figure 3). Age and FA in 10 white matter tracts were modelled as manifest variables. See Supplementary Table 8 for parameter estimates. Residual covariances between cardiovascular factors, lesion burden measures and age were allowed but are not shown for simplicity. Abbreviations: uncinate fasciculus (UNC), superior longitudinal fasciculus (SLF), inferior fronto-occipital fasciculus (IFOF), anterior thalamic radiations (ATR), cerebrospinal tract (CST), forceps major (FMaj), forceps minor (FMin), dorsal cingulate gyrus (CG), ventral cingulate gyrus (CH), inferior longitudinal fasciculus (ILF).

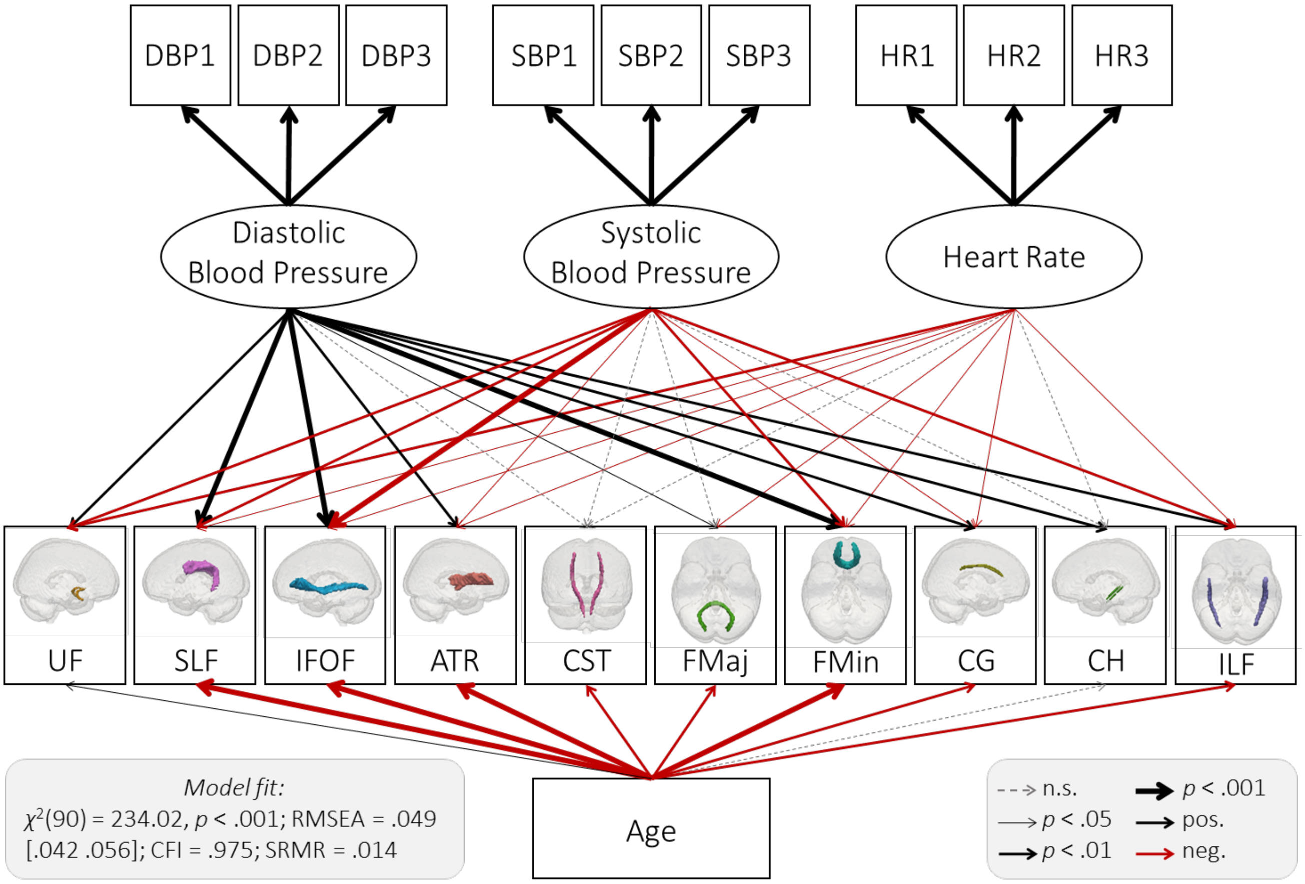

Supplementary Figure 2. Path Model of the Relationship between Cardiovascular Health and MK. Diastolic blood pressure, systolic blood pressure and heart rate were modelled as latent variables (Figure 3). Age and MK in 10 white matter tracts were modelled as manifest variables. See Supplementary Table 10 for parameter estimates.

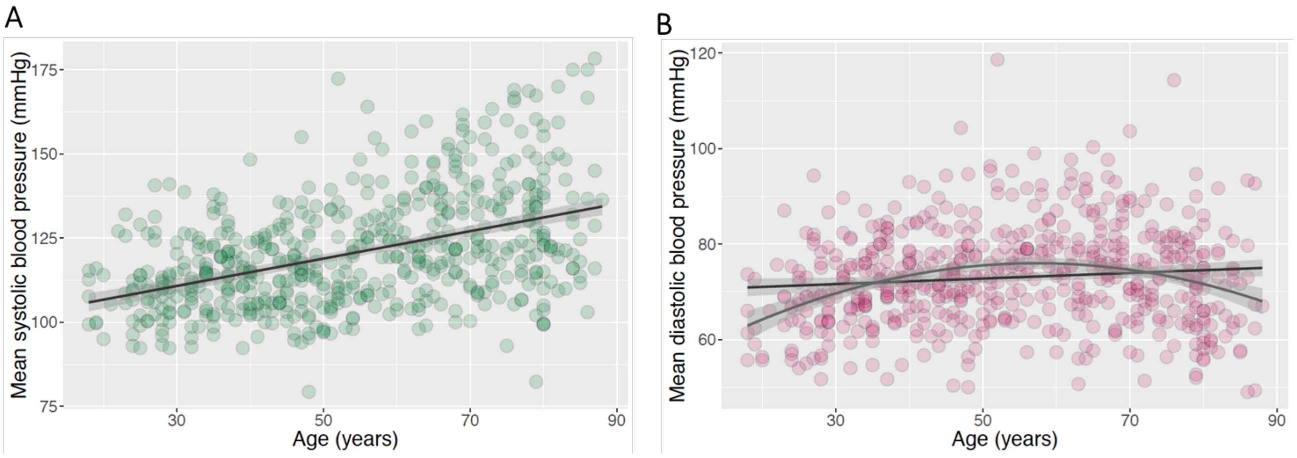

Supplementary Figure 3. Systolic and Diastolic Blood Pressure in the Cam-CAN Cohort. Scatter plots of mean systolic (Panel A) and diastolic blood pressure (Panel B) by age with linear regression lines shown in black and quadratic regression lines shown in grey.

Supplementary Tables

Supplementary Table 1. Regression Estimates for the Model Estimating White Matter Lesion Burden for different thresholds κ

Path			Estimate	SE	*z*	*p*	Standardized Estimate
				κ = 0.1			
TLV	~	DBP	-0.32	0.09	-3.45	.001	-0.32
TLV	~	SBP	0.32	0.11	2.94	.003	0.32
TLV	~	HR	0.20	0.06	3.15	.002	0.20
TLV	~	age	0.43	0.06	7.74	< .001	0.43
TLN	~	DBP	0.00	0.11	0.03	.977	0.00
TLN	~	SBP	0.08	0.10	0.77	.441	0.08
TLN	~	HR	0.09	0.06	1.66	.096	0.09
TLN	~	age	0.43	0.06	7.29	< .001	0.43
				κ = 0.3			
TLV	~	DBP	-0.33	0.10	-3.34	.001	-0.33
TLV	~	SBP	0.34	0.12	2.80	.005	0.34
TLV	~	HR	0.20	0.07	2.81	.005	0.20
TLV	~	age	0.37	0.06	6.38	< .001	0.37
TLN	~	DBP	-0.21	0.09	-2.26	.024	-0.20
TLN	~	SBP	0.30	0.15	2.03	.043	0.30
TLN	~	HR	0.06	0.04	1.48	.139	0.06
TLN	~	age	0.55	0.06	9.62	< .001	0.54
				κ = 0.5			
TLV	~	DBP	-0.34	0.10	-3.35	.001	-0.34
TLV	~	SBP	0.35	0.12	2.84	.004	0.35
TLV	~	HR	0.20	0.07	2.77	.006	0.20
TLV	~	age	0.34	0.06	5.93	< .001	0.34
TLN	~	DBP	-0.24	0.09	-2.62	.009	-0.24
TLN	~	SBP	0.32	0.16	2.05	.040	0.32
TLN	~	HR	0.11	0.04	2.64	.008	0.11
TLN	~	age	0.54	0.06	9.19	< .001	0.54
				κ = 0.7			
TLV	~	DBP	-0.35	0.10	-3.30	.001	-0.35
TLV	~	SBP	0.35	0.12	2.81	.005	0.35
TLV	~	HR	0.20	0.07	2.73	.006	0.20
TLV	~	age	0.32	0.06	5.56	< .001	0.32
TLN	~	DBP	-0.23	0.09	-2.54	.011	-0.23
TLN	~	SBP	0.28	0.15	1.86	.063	0.28
TLN	~	HR	0.12	0.04	2.87	.004	0.12
TLN	~	age	0.57	0.06	9.57	< .001	0.57
				κ = 0.9			
TLV	~	DBP	-0.35	0.11	-3.26	.001	-0.35
TLV	~	SBP	0.35	0.13	2.78	.005	0.35
TLV	~	HR	0.20	0.08	2.72	.007	0.20
TLV	~	age	0.31	0.06	5.28	< .001	0.31
TLN	~	DBP	-0.27	0.09	-3.00	.003	-0.27
TLN	~	SBP	0.30	0.15	2.05	.041	0.30
TLN	~	HR	0.13	0.04	3.30	.001	0.13
TLN	~	age	0.57	0.06	9.76	< .001	0.56

Note. DBP = diastolic blood pressure; SBP = systolic blood pressure; HR = heart rate; TLV = total lesion volume; TLN = total lesion number; Model fit: κ = 0.1: $\chi^2(42) = 166.36$, $p < .001$; RMSEA = .067 [.058 .076]; CFI = .954; SRMR = .021; κ = 0.3: $\chi^2(42) = 168.28$, $p < .001$; RMSEA = .067 [.058 .076]; CFI = .953; SRMR = .021; κ = 0.5: $\chi^2(42) = 165.15$, $p < .001$; RMSEA = .066 [.058 .075]; CFI = .954; SRMR = .022; κ = 0.7: $\chi^2(42) = 170.38$, $p < .001$; RMSEA = .068 [.059 .077]; CFI = .952; SRMR = .021; κ = 0.9: $\chi^2(42) = 169.84$, $p < .001$; RMSEA = .068 [.059 .077]; CFI = .952; SRMR = .021

Supplementary Table 2. Latent Variable Loadings for the Three-Factor Measurement Model

Path			Estimate	SE	*z*	*p*	Standardized Estimate
DPB	=~	DBP1	0.88	0.04	20.39	< .001	0.88
DPB	=~	DBP2	0.95	0.03	27.16	< .001	0.95
DPB	=~	DBP3	0.95	0.03	27.89	< .001	0.95
SPB	=~	SBP1	0.88	0.05	18.26	< .001	0.88
SPB	=~	SBP2	0.96	0.04	26.60	< .001	0.96
SPB	=~	SBP3	0.93	0.04	24.63	< .001	0.93
HR	=~	HR1	0.96	0.04	27.31	< .001	0.96
HR	=~	HR2	0.95	0.04	26.51	< .001	0.95
HR	=~	HR3	0.92	0.04	24.04	< .001	0.92

Supplementary Table 3. Latent Variable Loadings for the Single-Factor Measurement Model

Path			Estimate	SE	*z*	*p*	Standardized Estimate
CV	=~	DBP1	0.88	0.04	21.07	< .001	0.88
CV	=~	DBP2	0.94	0.04	26.35	< .001	0.94
CV	=~	DBP3	0.93	0.03	27.22	< .001	0.94
CV	=~	SBP1	0.66	0.04	15.87	< .001	0.66
CV	=~	SBP2	0.69	0.04	16.96	< .001	0.70
CV	=~	SBP3	0.69	0.04	15.95	< .001	0.69
CV	=~	HR1	0.28	0.04	6.36	< .001	0.28
CV	=~	HR2	0.29	0.05	6.18	< .001	0.29
CV	=~	HR3	0.28	0.05	6.16	< .001	0.28

Note. CV = single cardiovascular factor. Model fit statistics: $\chi^2(27) = 984.28$, $p < .001$; RMSEA = .247 [.240 .255]; CFI = .474; SRMR = .218

Supplementary Table 4. Regression Estimates for the Model Estimating White Matter Lesion Burden from Diastolic Blood Pressure only

Path			Estimate	SE	*z*	*p*	Standardized Estimate
TLV	~	DBP	-0.03	0.05	-0.61	.542	-0.03
TLN	~	DBP	0.03	0.06	0.47	.640	0.03

Note. Model fit statistics: $\chi^2(4) = 4.91$, $p = .297$; RMSEA = .019 [.000 .053]; CFI = .998; SRMR = .013

Supplementary Table 5. Regression Estimates for the Model Estimating White Matter Lesion Burden from Pulse Pressure and Systolic Blood pressure

Path			Estimate	SE	*z*	*p*	Standardized Estimate
TLV	~	PP	0.39	0.12	3.25	.001	0.39
TLV	~	SBP	-0.20	0.10	-1.98	.048	-0.20
TLV	~	HR	0.20	0.07	2.68	.007	0.20
TLV	~	age	0.33	0.06	5.75	< .001	0.33
TLN	~	PP	0.25	0.10	2.50	.012	0.25
TLN	~	SBP	-0.08	0.08	-0.99	.322	-0.08
TLN	~	HR	0.11	0.04	2.80	.005	0.11
TLN	~	age	0.58	0.06	10.12	< .001	0.57

Note. PP = pulse pressure; Model fit statistics: $\chi^2(39) = 59.38$, $p = .019$; RMSEA = .028 [.015 .039]; CFI = .993; SRMR = .022

Supplementary Table 6. Regression Estimates for the Model Estimating White Matter Lesion Burden from Pulse Pressure and Diastolic Blood pressure

Path			Estimate	SE	*z*	*p*	Standardized Estimate
TLV	~	PP	0.24	0.09	2.80	.005	0.24
TLV	~	DBP	-0.12	0.06	-2.01	.045	-0.12
TLV	~	HR	0.20	0.07	2.68	.007	0.20
TLV	~	age	0.33	0.06	5.77	< .001	0.34
TLN	~	PP	0.20	0.11	1.79	.074	0.20
TLN	~	DBP	-0.05	0.05	-1.03	.304	-0.05
TLN	~	HR	0.11	0.04	2.81	.005	0.11
TLN	~	age	0.58	0.06	10.03	< .001	0.57

Note. Model fit statistics: $\chi^2(39) = 56.58$, $p = .034$; RMSEA = .026 [.012 .038]; CFI = .992; SRMR = .021

Supplementary Table 7. Approximate R^2 of Cardiovascular Health and Age on FA, MD and MK

Tract	FA	MD	MK
UF	0.16	0.36	0.08
SLF	0.17	0.47	0.23
IFOF	0.32	0.38	0.26
ATR	0.25	0.56	0.19
CST	0.11	0.25	0.13
FMaj	0.12	0.21	0.13
FMin	0.51	0.54	0.28
CG	0.05	0.04	0.11
CH	0.02	0.06	0.04
ILF	0.25	0.21	0.09

Supplementary Table 8. Regression Estimates for the Model Estimating FA

Path			Estimate	SE	*z*	*p*	Standardized Estimate
UF	~	DBP	0.19	0.07	2.77	.006	0.19
UF	~	SBP	-0.26	0.08	-3.43	.001	-0.26
UF	~	HR	-0.15	0.04	-3.46	.001	-0.15
UF	~	age	-0.23	0.05	-4.96	< .001	-0.23
SLF	~	DBP	0.05	0.08	0.69	.490	0.05
SLF	~	SBP	-0.13	0.09	-1.45	.148	-0.13
SLF	~	HR	0.01	0.05	0.15	.882	0.01
SLF	~	age	-0.35	0.05	-6.93	< .001	-0.35
IFOF	~	DBP	0.14	0.06	2.17	.030	0.14
IFOF	~	SBP	-0.20	0.07	-2.79	.005	-0.20
IFOF	~	HR	-0.09	0.04	-2.36	.018	-0.09
IFOF	~	age	-0.46	0.05	-9.85	< .001	-0.46
ATR	~	DBP	0.18	0.06	2.78	.005	0.18
ATR	~	SBP	-0.15	0.08	-1.94	.052	-0.15
ATR	~	HR	-0.15	0.04	-3.60	< .001	-0.15
ATR	~	age	-0.40	0.05	-8.46	< .001	-0.40
CST	~	DBP	-0.05	0.07	-0.69	.493	-0.05
CST	~	SBP	-0.04	0.08	-0.45	.650	-0.04
CST	~	HR	0.01	0.04	0.22	.825	0.01
CST	~	age	-0.30	0.05	-6.00	< .001	-0.30
FMaj	~	DBP	0.11	0.07	1.51	.131	0.11
FMaj	~	SBP	-0.12	0.08	-1.46	.144	-0.12
FMaj	~	HR	-0.03	0.04	-0.64	.522	-0.03
FMaj	~	age	-0.29	0.05	-5.64	< .001	-0.29
FMin	~	DBP	0.12	0.06	2.08	.038	0.12
FMin	~	SBP	-0.09	0.06	-1.40	.162	-0.09
FMin	~	HR	-0.07	0.03	-2.16	.031	-0.07
FMin	~	age	-0.67	0.04	-16.97	< .001	-0.67
CG	~	DBP	0.06	0.08	0.75	.456	0.06
CG	~	SBP	-0.04	0.09	-0.39	.699	-0.04
CG	~	HR	-0.10	0.05	-2.25	.025	-0.10
CG	~	age	-0.19	0.05	-3.45	.001	-0.19
CH	~	DBP	-0.00	0.07	-0.04	.968	-0.00
CH	~	SBP	-0.04	0.08	-0.47	.639	-0.04
CH	~	HR	0.04	0.04	1.00	.318	0.04
CH	~	age	-0.12	0.05	-2.36	.018	-0.12
ILF	~	DBP	0.15	0.07	2.14	.032	0.15
ILF	~	SBP	-0.22	0.08	-2.85	.004	-0.22
ILF	~	HR	-0.02	0.04	-0.62	.538	-0.02
ILF	~	age	-0.40	0.05	-8.40	< .001	-0.40

Supplementary Table 9. Regression Estimates for the Model Estimating MD

Path			Estimate	SE	*z*	*p*	Standardized Estimate
UF	~	DBP	-0.36	0.07	-5.19	< .001	-0.36
UF	~	SBP	0.38	0.07	5.28	< .001	0.38
UF	~	HR	0.17	0.04	4.43	< .001	0.17
UF	~	age	0.38	0.04	9.02	< .001	0.38
SLF	~	DBP	-0.3	0.06	-4.95	< .001	-0.3
SLF	~	SBP	0.26	0.06	4.12	< .001	0.26
SLF	~	HR	0.11	0.04	2.76	0.006	0.11
SLF	~	age	0.55	0.04	13.37	< .001	0.55
IFOF	~	DBP	-0.24	0.06	-3.96	< .001	-0.24
IFOF	~	SBP	0.22	0.06	3.58	< .001	0.22
IFOF	~	HR	0.09	0.04	2.18	0.03	0.09
IFOF	~	age	0.51	0.05	10.99	< .001	0.51
ATR	~	DBP	-0.24	0.05	-4.94	< .001	-0.24
ATR	~	SBP	0.22	0.06	3.85	< .001	0.22
ATR	~	HR	0.12	0.03	3.63	< .001	0.12
ATR	~	age	0.63	0.04	17.45	< .001	0.63
CST	~	DBP	-0.22	0.08	-2.87	0.004	-0.22
CST	~	SBP	0.15	0.08	1.85	0.065	0.15
CST	~	HR	0.07	0.04	1.77	0.076	0.07
CST	~	age	0.42	0.05	7.93	< .001	0.42
FMaj	~	DBP	-0.23	0.06	-4.11	< .001	-0.23
FMaj	~	SBP	0.22	0.07	3.35	0.001	0.22
FMaj	~	HR	0.03	0.05	0.7	0.484	0.03
FMaj	~	age	0.35	0.05	6.41	< .001	0.35
FMin	~	DBP	-0.25	0.05	-5.07	< .001	-0.25
FMin	~	SBP	0.23	0.06	4.07	< .001	0.23
FMin	~	HR	0.12	0.03	3.75	< .001	0.12
FMin	~	age	0.62	0.04	16.01	< .001	0.62
CG	~	DBP	-0.14	0.08	-1.79	0.073	-0.14
CG	~	SBP	0.14	0.1	1.47	0.143	0.14
CG	~	HR	0.02	0.03	0.56	0.572	0.02
CG	~	age	0.12	0.07	1.84	0.065	0.12
CH	~	DBP	-0.22	0.09	-2.37	0.018	-0.22
CH	~	SBP	0.15	0.1	1.44	0.15	0.15
CH	~	HR	0.05	0.03	1.41	0.158	0.05
CH	~	age	0.15	0.06	2.52	0.012	0.15
ILF	~	DBP	-0.21	0.05	-4.27	< .001	-0.21
ILF	~	SBP	0.21	0.05	4.42	< .001	0.21
ILF	~	HR	0.03	0.05	0.69	0.491	0.03
ILF	~	age	0.35	0.06	6.2	< .001	0.35

Supplementary Table 10. Regression Estimates for the Model Estimating MK

Path			Estimate	SE	*z*	*p*	Standardized Estimate
UF	~	DBP	0.38	0.11	3.38	.001	0.38
UF	~	SBP	-0.32	0.11	-2.97	.003	-0.32
UF	~	HR	-0.21	0.08	-2.60	.009	-0.21
UF	~	age	0.11	0.05	2.14	.032	0.11
SLF	~	DBP	0.33	0.09	3.68	< .001	0.33
SLF	~	SBP	-0.28	0.09	-3.10	.002	-0.28
SLF	~	HR	-0.12	0.05	-2.19	.029	-0.12
SLF	~	age	-0.31	0.06	-5.50	< .001	-0.31
IFOF	~	DBP	0.36	0.09	4.03	< .001	0.36
IFOF	~	SBP	-0.32	0.09	-3.48	< .001	-0.32
IFOF	~	HR	-0.14	0.06	-2.32	.021	-0.14
IFOF	~	age	-0.32	0.05	-6.07	< .001	-0.32
ATR	~	DBP	0.35	0.11	3.07	.002	0.35
ATR	~	SBP	-0.27	0.11	-2.51	.012	-0.27
ATR	~	HR	-0.17	0.08	-2.08	.038	-0.17
ATR	~	age	-0.24	0.05	-4.43	< .001	-0.24
CST	~	DBP	0.28	0.17	1.69	.091	0.28
CST	~	SBP	-0.20	0.16	-1.28	.200	-0.20
CST	~	HR	-0.17	0.12	-1.41	.159	-0.17
CST	~	age	-0.21	0.06	-3.30	.001	-0.21
FMaj	~	DBP	0.27	0.11	2.40	.017	0.27
FMaj	~	SBP	-0.21	0.12	-1.84	.065	-0.21
FMaj	~	HR	-0.14	0.07	-2.01	.044	-0.14
FMaj	~	age	-0.21	0.08	-2.74	.006	-0.21
FMin	~	DBP	0.35	0.09	3.84	< .001	0.35
FMin	~	SBP	-0.30	0.09	-3.21	.001	-0.30
FMin	~	HR	-0.17	0.07	-2.58	.010	-0.17
FMin	~	age	-0.34	0.05	-6.61	< .001	-0.34
CG	~	DBP	0.33	0.10	3.27	.001	0.33
CG	~	SBP	-0.23	0.10	-2.21	.027	-0.23
CG	~	HR	-0.14	0.06	-2.30	.022	-0.14
CG	~	age	-0.16	0.06	-2.86	.004	-0.16
CH	~	DBP	0.27	0.09	3.12	.002	0.27
CH	~	SBP	-0.13	0.09	-1.46	.145	-0.13
CH	~	HR	-0.05	0.06	-0.96	.335	-0.05
CH	~	age	0.05	0.06	0.97	.332	0.05
ILF	~	DBP	0.26	0.08	3.34	.001	0.26
ILF	~	SBP	-0.23	0.08	-2.83	.005	-0.23
ILF	~	HR	-0.10	0.05	-2.10	.036	-0.10
ILF	~	age	-0.15	0.05	-2.73	.006	-0.15

Supplementary Table 11. Regression Estimates for the Model Estimating MD from Diastolic Blood Pressure only

Path			Estimate	SE	*z*	*p*	Standardized Estimate
UF	~	DBP	0.00	0.05	0.01	.988	0.00
SLF	~	DBP	-0.02	0.04	-0.47	.641	-0.02
IFOF	~	DBP	0.00	0.04	0.12	.908	0.00
ATR	~	DBP	0.02	0.04	0.54	.588	0.02
CST	~	DBP	-0.04	0.04	-0.93	.353	-0.04
FMaj	~	DBP	-0.03	0.03	-0.83	.405	-0.03
FMin	~	DBP	0.02	0.04	0.48	.631	0.02
CG	~	DBP	-0.02	0.02	-1.07	.285	-0.02
CH	~	DBP	-0.08	0.04	-2.10	.036	-0.08
ILF	~	DBP	-0.01	0.03	-0.31	.754	-0.01

Note. Model fit: $\chi^2(20) = 16.91$, $p = .659$; RMSEA = .000 [.000 .027]; CFI = 1.000; SRMR = .004

Supplementary Table 12. Regression Estimates for the Model Estimating MD from Pulse Pressure and Systolic Blood Pressure

Path			Estimate	SE	*z*	*p*	Standardized Estimate
UF	~	PP	0.41	0.08	5.08	< .001	0.41
UF	~	SBP	-0.19	0.07	-2.68	.007	-0.19
UF	~	HR	0.16	0.04	4.38	< .001	0.16
UF	~	age	0.39	0.04	9.39	< .001	0.39
SLF	~	PP	0.35	0.07	4.83	< .001	0.35
SLF	~	SBP	-0.22	0.06	-3.54	< .001	-0.22
SLF	~	HR	0.10	0.04	2.71	.007	0.10
SLF	~	age	0.56	0.04	13.67	< .001	0.56
IFOF	~	PP	0.27	0.07	3.86	< .001	0.27
IFOF	~	SBP	-0.16	0.06	-2.45	.014	-0.16
IFOF	~	HR	0.09	0.04	2.13	.033	0.09
IFOF	~	age	0.51	0.05	11.22	< .001	0.51
ATR	~	PP	0.27	0.06	4.86	< .001	0.28
ATR	~	SBP	-0.16	0.05	-3.10	.002	-0.16
ATR	~	HR	0.12	0.03	3.58	< .001	0.12
ATR	~	age	0.64	0.04	17.87	< .001	0.64
CST	~	PP	0.26	0.09	2.92	.003	0.26
CST	~	SBP	-0.20	0.07	-2.97	.003	-0.20
CST	~	HR	0.07	0.04	1.76	.078	0.07
CST	~	age	0.43	0.05	8.17	< .001	0.43
FMaj	~	PP	0.27	0.07	4.10	< .001	0.27
FMaj	~	SBP	-0.15	0.05	-2.77	.006	-0.15
FMaj	~	HR	0.03	0.05	0.66	.507	0.03
FMaj	~	age	0.35	0.05	6.58	< .001	0.35
FMin	~	PP	0.29	0.06	5.05	< .001	0.29
FMin	~	SBP	-0.17	0.05	-3.26	.001	-0.17
FMin	~	HR	0.12	0.03	3.71	< .001	0.12
FMin	~	age	0.63	0.04	16.39	< .001	0.63
CG	~	PP	0.16	0.09	1.81	.070	0.16
CG	~	SBP	-0.08	0.04	-2.35	.019	-0.08
CG	~	HR	0.01	0.03	0.52	.603	0.01
CG	~	age	0.13	0.06	1.96	.049	0.13
CH	~	PP	0.26	0.11	2.44	.015	0.26
CH	~	SBP	-0.20	0.07	-3.03	.002	-0.20
CH	~	HR	0.05	0.03	1.40	.162	0.05
CH	~	age	0.16	0.06	2.68	.007	0.16
ILF	~	PP	0.24	0.06	4.16	< .001	0.24
ILF	~	SBP	-0.13	0.05	-2.36	.018	-0.13
ILF	~	HR	0.03	0.05	0.65	.515	0.03
ILF	~	age	0.36	0.06	6.36	< .001	0.36

Note. Model fit statistics: $\chi^2(87) = 90.84$, $p = .368$; RMSEA = .008 [.000 .022]; CFI = .999; SRMR = .013

Supplementary Table 13. Regression Estimates for the Model Estimating MD from Pulse Pressure and Diastolic Blood Pressure

Path			Estimate	SE	*z*	*p*	Standardized Estimate
UF	~	PP	0.27	0.05	5.21	< .001	0.27
UF	~	DBP	-0.12	0.04	-2.70	.007	-0.12
UF	~	HR	0.16	0.04	4.38	< .001	0.16
UF	~	age	0.39	0.04	9.40	< .001	0.39
SLF	~	PP	0.19	0.05	4.03	< .001	0.19
SLF	~	DBP	-0.13	0.04	-3.58	< .001	-0.13
SLF	~	HR	0.10	0.04	2.72	.007	0.10
SLF	~	age	0.56	0.04	13.69	< .001	0.56
IFOF	~	PP	0.15	0.04	3.54	< .001	0.15
IFOF	~	DBP	-0.10	0.04	-2.47	.013	-0.10
IFOF	~	HR	0.09	0.04	2.14	.033	0.09
IFOF	~	age	0.51	0.05	11.24	< .001	0.51
ATR	~	PP	0.15	0.04	3.79	< .001	0.16
ATR	~	DBP	-0.10	0.03	-3.12	.002	-0.10
ATR	~	HR	0.12	0.03	3.58	< .001	0.12
ATR	~	age	0.64	0.04	17.91	< .001	0.64
CST	~	PP	0.11	0.06	1.83	.068	0.11
CST	~	DBP	-0.12	0.04	-2.96	.003	-0.12
CST	~	HR	0.07	0.04	1.76	.079	0.07
CST	~	age	0.43	0.05	8.17	< .001	0.43
FMaj	~	PP	0.16	0.05	3.33	.001	0.16
FMaj	~	DBP	-0.09	0.03	-2.77	.006	-0.09
FMaj	~	HR	0.03	0.05	0.66	.509	0.03
FMaj	~	age	0.35	0.05	6.61	< .001	0.35
FMin	~	PP	0.16	0.04	4.04	< .001	0.16
FMin	~	DBP	-0.10	0.03	-3.28	.001	-0.10
FMin	~	HR	0.12	0.03	3.72	< .001	0.12
FMin	~	age	0.63	0.04	16.41	< .001	0.63
CG	~	PP	0.10	0.07	1.46	.146	0.10
CG	~	DBP	-0.05	0.02	-2.35	.019	-0.05
CG	~	HR	0.01	0.03	0.53	.599	0.01
CG	~	age	0.13	0.06	1.96	.050	0.12
CH	~	PP	0.11	0.07	1.44	.151	0.11
CH	~	DBP	-0.13	0.04	-3.02	.003	-0.13
CH	~	HR	0.05	0.03	1.40	.162	0.05
CH	~	age	0.16	0.06	2.67	.008	0.16
ILF	~	PP	0.15	0.03	4.38	< .001	0.15
ILF	~	DBP	-0.08	0.03	-2.39	.017	-0.08
ILF	~	HR	0.03	0.05	0.65	.513	0.03
ILF	~	age	0.36	0.06	6.38	< .001	0.36

Note. Model fit statistics: $\chi^2(87) = 87.81$, $p = .455$; RMSEA = .004 [.000 .020]; CFI = 1.000; SRMR = .013

Supplementary Table 14. Regression Estimates for the Model Estimating the Relationship between Exercise and MD

Path			Estimate	SE	*z*	*p*	Standardized Estimate
UF	~	DBP	-0.32	0.09	-3.56	< .001	-0.30
UF	~	SBP	0.37	0.09	4.23	< .001	0.35
UF	~	HR	0.15	0.04	3.88	< .001	0.14
UF	~	age	0.30	0.05	6.25	< .001	0.28
UF	~	exercise home	-0.02	0.04	-0.42	.674	-0.01
UF	~	exercise leisure	-0.05	0.03	-1.59	.112	-0.05
UF	~	exercise work	-0.13	0.03	-4.02	< .001	-0.12
UF	~	exercise commute	0.01	0.03	0.27	.790	0.01
SLF	~	DBP	-0.30	0.10	-2.95	.003	-0.28
SLF	~	SBP	0.27	0.10	2.73	.006	0.25
SLF	~	HR	0.11	0.04	2.50	.012	0.10
SLF	~	age	0.50	0.05	10.35	< .001	0.47
SLF	~	exercise home	-0.06	0.03	-1.75	.079	-0.05
SLF	~	exercise leisure	-0.01	0.03	-0.48	.631	-0.01
SLF	~	exercise work	-0.05	0.03	-1.53	.127	-0.04
SLF	~	exercise commute	-0.01	0.02	-0.53	.594	-0.01
IFOF	~	DBP	-0.25	0.11	-2.34	.019	-0.24
IFOF	~	SBP	0.25	0.10	2.41	.016	0.24
IFOF	~	HR	0.09	0.05	1.96	.050	0.09
IFOF	~	age	0.45	0.05	8.62	< .001	0.44
IFOF	~	exercise home	-0.04	0.03	-1.39	.164	-0.04
IFOF	~	exercise leisure	-0.05	0.03	-1.60	.109	-0.04
IFOF	~	exercise work	-0.06	0.02	-2.53	.011	-0.06
IFOF	~	exercise commute	0.01	0.02	0.48	.628	0.01
ATR	~	DBP	-0.20	0.04	-4.69	< .001	-0.19
ATR	~	SBP	0.20	0.05	3.96	< .001	0.19
ATR	~	HR	0.11	0.03	3.56	< .001	0.11
ATR	~	age	0.60	0.04	15.67	< .001	0.58
ATR	~	exercise home	-0.03	0.03	-1.09	.274	-0.03
ATR	~	exercise leisure	0.02	0.03	0.61	.544	0.02
ATR	~	exercise work	-0.09	0.03	-2.81	.005	-0.08
ATR	~	exercise commute	0.04	0.03	1.39	.165	0.04
CST	~	DBP	-0.24	0.13	-1.87	.061	-0.23
CST	~	SBP	0.19	0.12	1.53	.125	0.18
CST	~	HR	0.07	0.05	1.48	.139	0.07
CST	~	age	0.34	0.06	5.49	< .001	0.34
CST	~	exercise home	-0.06	0.04	-1.63	.103	-0.05
CST	~	exercise leisure	-0.06	0.03	-2.02	.043	-0.06
CST	~	exercise work	-0.08	0.03	-2.74	.006	-0.07
CST	~	exercise commute	-0.02	0.02	-0.80	.426	-0.02
FMaj	~	DBP	-0.21	0.06	-3.48	< .001	-0.20
FMaj	~	SBP	0.20	0.06	3.15	.002	0.20
FMaj	~	HR	0.03	0.05	0.68	.498	0.03
FMaj	~	age	0.32	0.05	5.88	< .001	0.32
FMaj	~	exercise home	-0.03	0.03	-1.10	.270	-0.03
FMaj	~	exercise leisure	0.04	0.04	0.97	.330	0.04
FMaj	~	exercise work	-0.05	0.04	-1.22	.224	-0.05

Path			Estimate	SE	*z*	*p*	Standardized Estimate
FMaj	~	exercise commute	0.01	0.03	0.31	.759	0.01
FMin	~	DBP	-0.23	0.05	-4.21	< .001	-0.21
FMin	~	SBP	0.22	0.06	3.86	< .001	0.21
FMin	~	HR	0.12	0.03	3.50	< .001	0.11
FMin	~	age	0.57	0.04	14.20	< .001	0.55
FMin	~	exercise home	-0.03	0.03	-1.03	.303	-0.03
FMin	~	exercise leisure	-0.02	0.03	-0.89	.374	-0.02
FMin	~	exercise work	-0.08	0.03	-2.48	.013	-0.07
FMin	~	exercise commute	0.00	0.03	0.14	.885	0.00
CG	~	DBP	-0.12	0.06	-2.15	.032	-0.12
CG	~	SBP	0.13	0.07	1.81	.071	0.13
CG	~	HR	0.01	0.03	0.26	.797	0.01
CG	~	age	0.09	0.07	1.21	.225	0.09
CG	~	exercise home	-0.04	0.04	-0.90	.367	-0.04
CG	~	exercise leisure	-0.04	0.03	-1.69	.090	-0.04
CG	~	exercise work	-0.04	0.01	-2.96	.003	-0.04
CG	~	exercise commute	-0.03	0.03	-1.09	.275	-0.03
CH	~	DBP	-0.22	0.11	-1.89	.059	-0.22
CH	~	SBP	0.17	0.12	1.47	.141	0.17
CH	~	HR	0.04	0.04	1.04	.299	0.04
CH	~	age	0.07	0.07	1.05	.293	0.08
CH	~	exercise home	-0.03	0.04	-0.68	.499	-0.03
CH	~	exercise leisure	-0.04	0.04	-0.98	.325	-0.04
CH	~	exercise work	-0.10	0.03	-3.13	.002	-0.09
CH	~	exercise commute	-0.02	0.03	-0.71	.480	-0.02
ILF	~	DBP	-0.22	0.08	-2.67	.008	-0.21
ILF	~	SBP	0.23	0.08	2.98	.003	0.22
ILF	~	HR	0.03	0.05	0.63	.528	0.03
ILF	~	age	0.31	0.06	5.36	< .001	0.31
ILF	~	exercise home	-0.00	0.03	-0.17	.868	-0.00
ILF	~	exercise leisure	-0.05	0.03	-1.74	.082	-0.05
ILF	~	exercise work	-0.05	0.02	-1.94	.053	-0.04
ILF	~	exercise commute	0.00	0.02	0.15	.878	0.00
DBP	~	exercise home	-0.00	0.05	-0.04	.967	-0.00
DBP	~	exercise leisure	-0.03	0.05	-0.68	.495	-0.03
DBP	~	exercise work	0.19	0.06	3.39	.001	0.19
DBP	~	exercise commute	-0.17	0.05	-3.21	.001	-0.17
SBP	~	exercise home	-0.11	0.05	-2.13	.033	-0.10
SBP	~	exercise leisure	0.03	0.05	0.56	.574	0.03
SBP	~	exercise work	-0.02	0.06	-0.42	.672	-0.02
SBP	~	exercise commute	-0.21	0.04	-4.66	< .001	-0.20
HR	~	exercise home	0.04	0.04	0.89	.372	0.04
HR	~	exercise leisure	-0.05	0.07	-0.72	.472	-0.05
HR	~	exercise work	-0.02	0.04	-0.44	.662	-0.02
HR	~	exercise commute	-0.13	0.04	-3.34	.001	-0.13

Supplementary Table 15. Regression Estimates for the Model Estimating effects of BMI on MD

Path			Estimate	SE	*z*	*p*	Standardized Estimate
UF	~	DBP	-0.31	0.09	-3.59	< .001	-0.32
UF	~	SBP	0.35	0.09	3.94	< .001	0.35
UF	~	HR	0.16	0.04	4.15	< .001	0.16
UF	~	age	0.42	0.04	10.28	< .001	0.42
UF	~	BMI	-0.05	0.04	-1.31	.191	-0.05
SLF	~	DBP	-0.30	0.10	-3.16	.002	-0.32
SLF	~	SBP	0.28	0.10	2.85	.004	0.29
SLF	~	HR	0.10	0.04	2.69	.007	0.11
SLF	~	age	0.56	0.05	12.27	< .001	0.57
SLF	~	BMI	-0.01	0.03	-0.40	.688	-0.01
IFOF	~	DBP	-0.24	0.11	-2.28	.022	-0.25
IFOF	~	SBP	0.25	0.11	2.31	.021	0.26
IFOF	~	HR	0.09	0.04	2.17	.030	0.10
IFOF	~	age	0.52	0.05	10.19	< .001	0.53
IFOF	~	BMI	-0.06	0.03	-1.70	.089	-0.06
ATR	~	DBP	-0.19	0.04	-4.68	< .001	-0.20
ATR	~	SBP	0.18	0.05	3.93	< .001	0.19
ATR	~	HR	0.11	0.03	3.61	< .001	0.12
ATR	~	age	0.67	0.03	19.90	< .001	0.69
ATR	~	BMI	-0.07	0.03	-2.24	.025	-0.07
CST	~	DBP	-0.24	0.12	-1.89	.059	-0.25
CST	~	SBP	0.19	0.13	1.50	.133	0.20
CST	~	HR	0.08	0.05	1.73	.083	0.08
CST	~	age	0.43	0.05	7.80	< .001	0.43
CST	~	BMI	-0.05	0.04	-1.08	.280	-0.05
FMaj	~	DBP	-0.19	0.05	-3.50	< .001	-0.20
FMaj	~	SBP	0.20	0.06	3.34	.001	0.21
FMaj	~	HR	0.03	0.04	0.71	.480	0.03
FMaj	~	age	0.38	0.05	7.16	< .001	0.38
FMaj	~	BMI	-0.08	0.03	-2.26	.024	-0.08
FMin	~	DBP	-0.24	0.05	-4.97	< .001	-0.26
FMin	~	SBP	0.22	0.05	4.25	< .001	0.23
FMin	~	HR	0.12	0.03	3.77	< .001	0.12
FMin	~	age	0.63	0.04	17.31	< .001	0.64
FMin	~	BMI	0.01	0.03	0.44	.661	0.01
CG	~	DBP	-0.11	0.05	-2.25	.024	-0.12
CG	~	SBP	0.12	0.06	1.91	.056	0.12
CG	~	HR	0.01	0.03	0.38	.702	0.01
CG	~	age	0.14	0.05	2.57	.010	0.14
CG	~	BMI	-0.01	0.02	-0.81	.418	-0.01
CH	~	DBP	-0.21	0.11	-1.86	.063	-0.22
CH	~	SBP	0.16	0.12	1.38	.169	0.16
CH	~	HR	0.05	0.04	1.32	.188	0.05
CH	~	age	0.17	0.05	3.17	.002	0.17
CH	~	BMI	-0.06	0.04	-1.61	.108	-0.06
ILF	~	DBP	-0.20	0.08	-2.58	.010	-0.21
ILF	~	SBP	0.22	0.08	2.90	.004	0.23
ILF	~	HR	0.04	0.05	0.84	.401	0.04
ILF	~	age	0.37	0.06	6.34	< .001	0.37
ILF	~	BMI	-0.08	0.03	-2.53	.011	-0.08
DBP	~	BMI	0.30	0.05	6.61	< .001	0.29
SBP	~	BMI	0.24	0.05	5.21	< .001	0.23
HR	~	BMI	0.17	0.04	3.77	< .001	0.17

Supplementary Table 16. Regression Estimates for the Model Estimating effects of BMI only on MD

Path			Estimate	SE	*z*	*p*	Standardized Estimate
UF	~	BMI	0.08	0.05	1.84	.066	0.08
SLF	~	BMI	0.13	0.04	3.21	.001	0.13
IFOF	~	BMI	0.09	0.04	2.17	.030	0.09
ATR	~	BMI	0.12	0.04	2.77	.006	0.12
CST	~	BMI	0.06	0.04	1.29	.197	0.06
FMaj	~	BMI	0.02	0.03	0.63	.531	0.02
FMin	~	BMI	0.18	0.04	4.13	< .001	0.18
CG	~	BMI	0.02	0.02	1.21	.227	0.02
CH	~	BMI	-0.03	0.03	-0.88	.380	-0.03
ILF	~	BMI	0.03	0.03	0.85	.397	0.03

Note. Model is just identified.

Supplementary Table 17. Regression Estimates for the Model Estimating effects of Social and Lifestyle factors on lesion burden

Path			Estimate	SE	*z*	*p*	Standardized Estimate
TLV	~	DBP	-0.35	0.11	-3.26	.001	-0.36
TLV	~	SBP	0.35	0.12	2.83	.005	0.35
TLV	~	HR	0.19	0.07	2.74	.006	0.20
TLV	~	age	0.31	0.06	5.39	< .001	0.31
TLV	~	alcohol	0.05	0.06	0.86	.392	0.05
TLV	~	smoking	0.05	0.05	0.85	.398	0.05
TLV	~	social class	-0.04	0.06	-0.58	.564	-0.04
TLV	~	education	-0.01	0.08	-0.14	.885	-0.01
TLN	~	DBP	-0.24	0.09	-2.64	.008	-0.24
TLN	~	SBP	0.29	0.15	1.90	.057	0.28
TLN	~	HR	0.11	0.04	2.62	.009	0.11
TLN	~	age	0.54	0.06	9.29	< .001	0.53
TLN	~	alcohol	0.10	0.05	2.17	.030	0.10
TLN	~	smoking	-0.02	0.05	-0.44	.657	-0.02
TLN	~	social class	0.02	0.04	0.46	.649	0.02
TLN	~	education	-0.08	0.06	-1.32	.186	-0.08

Note. Model fit statistics: $\chi^2(66) = 218.74$, $p < .001$; RMSEA = .059 [.051 .067]; CFI = .960; SRMR = .018

Supplementary Table 18. Regression Estimates for the Model Estimating effects of Social and Lifestyle factors on MD

Path			Estimate	SE	*z*	*p*	Standardized Estimate
UF	~	DBP	-0.36	0.07	-5.01	< .001	-0.36
UF	~	SBP	0.38	0.07	5.13	< .001	0.38
UF	~	HR	0.16	0.04	4.19	< .001	0.16
UF	~	age	0.38	0.04	8.71	< .001	0.38
UF	~	alcohol	0.04	0.04	1.02	.306	0.04
UF	~	smoking	-0.06	0.03	-1.82	.069	-0.06
UF	~	social class	-0.05	0.04	-1.18	.238	-0.05
UF	~	education	-0.00	0.04	-0.04	.964	-0.00
SLF	~	DBP	-0.31	0.06	-4.97	< .001	-0.31
SLF	~	SBP	0.27	0.07	4.17	< .001	0.27
SLF	~	HR	0.11	0.04	3.03	.002	0.11
SLF	~	age	0.54	0.04	12.62	< .001	0.54
SLF	~	alcohol	0.09	0.03	2.63	.009	0.09
SLF	~	smoking	0.01	0.03	0.27	.785	0.01
SLF	~	social class	-0.01	0.03	-0.21	.835	-0.01
SLF	~	education	0.01	0.04	0.15	.884	0.01
IFOF	~	DBP	-0.25	0.06	-4.05	< .001	-0.25
IFOF	~	SBP	0.23	0.06	3.67	< .001	0.23
IFOF	~	HR	0.09	0.04	2.42	.016	0.09
IFOF	~	age	0.50	0.05	10.41	< .001	0.50
IFOF	~	alcohol	0.08	0.03	2.35	.019	0.08
IFOF	~	smoking	-0.01	0.03	-0.23	.817	-0.01
IFOF	~	social class	-0.02	0.03	-0.77	.443	-0.02
IFOF	~	education	0.02	0.04	0.53	.593	0.02
ATR	~	DBP	-0.24	0.05	-4.94	< .001	-0.24
ATR	~	SBP	0.22	0.06	3.83	< .001	0.22
ATR	~	HR	0.12	0.03	3.73	< .001	0.12
ATR	~	age	0.63	0.04	17.44	< .001	0.63
ATR	~	alcohol	0.04	0.03	1.49	.135	0.04
ATR	~	smoking	-0.02	0.03	-0.82	.410	-0.02
ATR	~	social class	-0.01	0.03	-0.34	.735	-0.01
ATR	~	education	0.01	0.04	0.21	.836	0.01
CST	~	DBP	-0.22	0.08	-2.76	.006	-0.22
CST	~	SBP	0.15	0.08	1.74	.082	0.15
CST	~	HR	0.07	0.04	1.74	.082	0.07
CST	~	age	0.42	0.06	7.28	< .001	0.42
CST	~	alcohol	0.04	0.04	1.00	.315	0.04
CST	~	smoking	-0.03	0.04	-0.93	.354	-0.03
CST	~	social class	-0.02	0.03	-0.69	.488	-0.02
CST	~	education	-0.02	0.04	-0.40	.689	-0.02
FMaj	~	DBP	-0.24	0.06	-4.32	< .001	-0.24

Path			Estimate	SE	*z*	*p*	Standardized Estimate
FMaj	~	SBP	0.23	0.07	3.49	< .001	0.23
FMaj	~	HR	0.04	0.04	0.98	.326	0.04
FMaj	~	age	0.34	0.06	6.02	< .001	0.34
FMaj	~	alcohol	0.09	0.03	2.70	.007	0.09
FMaj	~	smoking	0.01	0.04	0.18	.856	0.01
FMaj	~	social class	-0.00	0.03	-0.05	.960	-0.00
FMaj	~	education	0.03	0.05	0.55	.583	0.03
FMin	~	DBP	-0.26	0.05	-5.10	< .001	-0.26
FMin	~	SBP	0.23	0.06	4.02	< .001	0.23
FMin	~	HR	0.13	0.03	4.01	< .001	0.13
FMin	~	age	0.60	0.04	14.94	< .001	0.60
FMin	~	alcohol	0.07	0.03	2.16	.030	0.07
FMin	~	smoking	0.01	0.03	0.25	.803	0.01
FMin	~	social class	0.00	0.03	0.07	.946	0.00
FMin	~	education	-0.02	0.04	-0.56	.577	-0.02
CG	~	DBP	-0.15	0.08	-1.75	.080	-0.15
CG	~	SBP	0.15	0.10	1.49	.137	0.15
CG	~	HR	0.02	0.03	0.78	.435	0.02
CG	~	age	0.12	0.07	1.59	.113	0.12
CG	~	alcohol	0.03	0.03	0.81	.420	0.03
CG	~	smoking	0.03	0.05	0.74	.458	0.03
CG	~	social class	-0.01	0.02	-0.64	.520	-0.01
CG	~	education	0.02	0.03	0.76	.446	0.02
CH	~	DBP	-0.23	0.10	-2.31	.021	-0.23
CH	~	SBP	0.15	0.11	1.43	.153	0.15
CH	~	HR	0.04	0.04	1.25	.213	0.04
CH	~	age	0.15	0.07	2.18	.029	0.15
CH	~	alcohol	0.06	0.03	1.78	.075	0.06
CH	~	smoking	-0.04	0.04	-0.84	.399	-0.04
CH	~	social class	-0.06	0.03	-2.10	.036	-0.06
CH	~	education	0.00	0.03	0.05	.958	0.00
ILF	~	DBP	-0.22	0.05	-4.55	< .001	-0.22
ILF	~	SBP	0.22	0.05	4.72	< .001	0.22
ILF	~	HR	0.04	0.04	0.91	.360	0.04
ILF	~	age	0.35	0.06	5.88	< .001	0.35
ILF	~	alcohol	0.09	0.03	3.12	.002	0.09
ILF	~	smoking	-0.00	0.04	-0.09	.928	-0.00
ILF	~	social class	-0.03	0.03	-1.13	.260	-0.03
ILF	~	education	0.05	0.05	0.95	.340	0.05

Note. Model fit statistics: $\chi^2(114) = 253.10$, $p < .001$; RMSEA = .043 [.036 .049]; CFI = .982; SRMR = .012

Supplementary References

de Groot, M., Vernooij, M. W., Klein, S., Ikram, M. A., Vos, F. M., Smith, S. M., ... & Andersson, J. L. 2013) Improving alignment in tract-based spatial statistics: evaluation and optimization of image registration. Neuroimage, 76, 400-411.
